# Idiopathic fourth ventricle outlet obstruction successfully treated by endoscopic third ventriculostomy: a case report

**DOI:** 10.1186/s40064-015-1368-x

**Published:** 2015-09-30

**Authors:** Yukitomo Ishi, Katsuyuki Asaoka, Hiroyuki Kobayashi, Hiroaki Motegi, Taku Sugiyama, Yuka Yokoyama, Sumire Echizenya, Koji Itamoto

**Affiliations:** Department of Neurosurgery, Teine Keijinkai Hospital, 1-40, Maeda 1-12, Teine-ku, Sapporo, 006-8555 Japan; Department of Neurosurgery, Hokkaido University Graduate School of Medicine, North 15 West 7, Kita-ku, Sapporo, 060-8638 Japan

**Keywords:** Hydrocephalus, Fourth ventricle outlet obstruction, Endoscopic third ventriculostomy, FVOO, ETV

## Abstract

**Introduction:**

Fourth ventricle outlet obstruction (FVOO) is a rare cause of obstructive hydrocephalus. We describe a case of idiopathic FVOO that was successfully treated with endoscopic third ventriculostomy (ETV).

**Case report:**

A 3-year old boy without any remarkable medical history presented with a headache and vomiting. Computed tomography (CT) images, which had incidentally been taken 2 years previously due to a minor head injury, showed no abnormality. Magnetic resonance imaging on admission showed tetra-ventricular hydrocephalus associated with the dilatation of the fourth ventricle outlets, without any obstructive lesions. However, CT ventriculography, involving contrast medium injection through a ventricular catheter, suggested mechanical obstruction of the cerebrospinal fluid (CSF) at the fourth ventricle outlets. Thus, the patient was diagnosed with FVOO and ETV was performed; the hydrocephalus was subsequently resolved. Although hydrocephalus recurred 1 year postoperatively, re-ETV for the highly stenosed fenestration successfully resolved this condition.

**Conclusions:**

ETV should be considered for FVOO treatment, particularly in idiopathic cases without CSF malabsorption.

## Background

Fourth ventricle outlet obstruction (FVOO) is an uncommon clinical condition that causes obstructive hydrocephalus. In FVOO, cerebrospinal fluid (CSF) is blocked at the fourth ventricle outlets by a membranous structure in the absence of any additional obstructive organic pathologies. Various terms for referring to FVOO have been used in previous reports, such as fourth ventricle/ventricular outlet obstruction (Ferrer and de Notaris 2013; Mohanty et al. 2008; Roth et al. 2012; Suehiro et al. 2000), fourth ventricular outflow obstruction (Karachi et al. 2003), membranous obstruction of the fourth ventricle outlet (Huang et al. 2001), obstruction of Magendie’s and Luschka’s foramina (Carpentier et al. 2001), obstruction of fourth ventricular exit (Choi et al. 1999) and primary obstruction of the fourth ventricle outlets (Longatti et al. 2009). Far distal obstructive hydrocephalus is a term that includes Dandy Walker or Arnold Chiari malformation, membranous obstruction or fourth ventricle and intercisternal external obstruction of the CSF (Oertel et al. 2010). The etiology and pathogenesis of FVOO are unclear, although some cases present with a history of meningitis or intraventricular hemorrhage. In the present report, we describe the case of childhood idiopathic FVOO without any remarkable medical history that was successfully treated by endoscopic third ventriculostomy (ETV), and also provide a review of the relevant literature.

## Case report

**Fig. 1:**
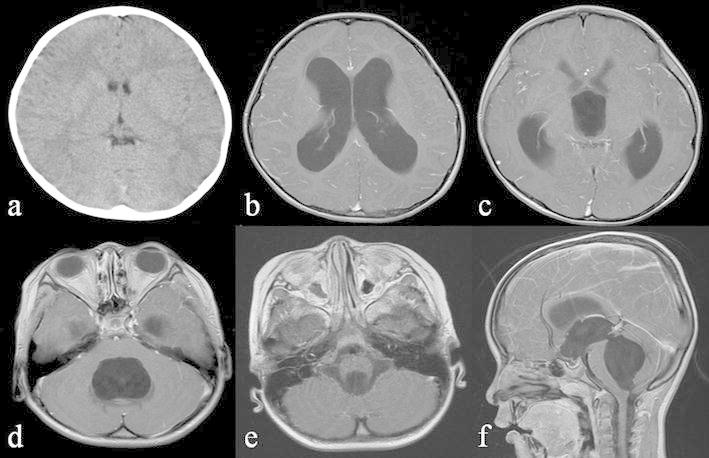
**a** Computed tomography images obtained to evaluate a head injury 2 years prior to admission, which did not indicate any apparent intracranial abnormality. **b**–**d** Contrast-enhanced T1-weighted image (WI) on admission indicating the dilatation of bilateral, third and fourth ventricles. **e**–**f** Contrast-enhanced T1-WI (*E* axial, *F* sagittal) indicating no obstructive lesions such as a brain tumor or cerebellar anomaly

**Fig. 2:**
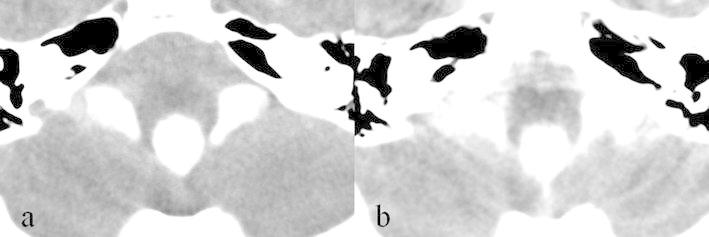
Computed tomography images after the injection of contrast medium through the ventricular catheter (**a** 1 h after injection, **b** 3 h after injection). Both images show delayed diffusion of contrast medium to the pre-pontine cistern

**Fig. 3:**
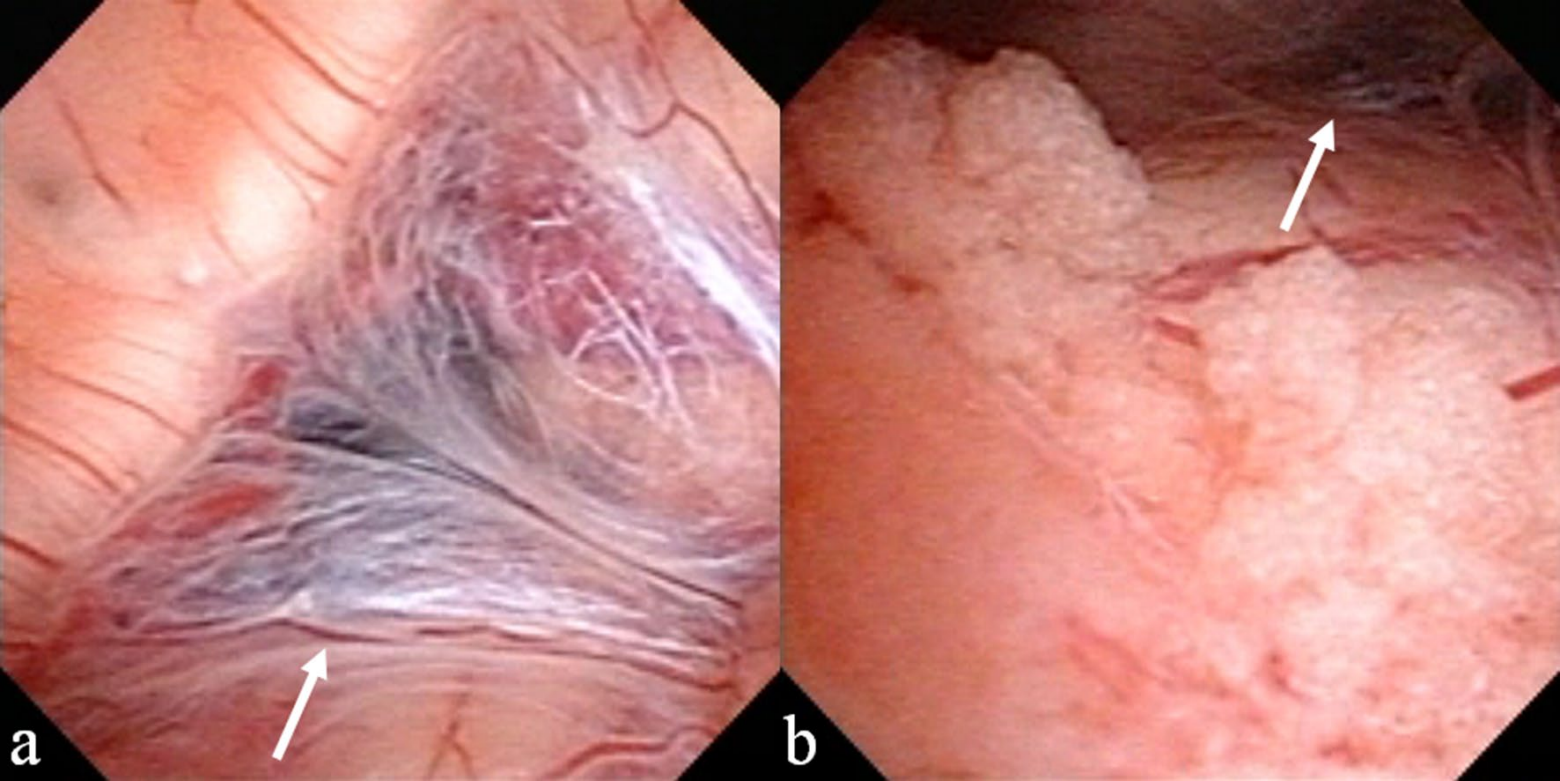
Intraoperative view of the foramina of Magendie (**a**) and Luschka (**b**) via neuroendoscopy. Membranous lesions (*arrow*) covering both foramina suggested the blockage of CSF flow in the fourth ventricle outlets

**Fig. 4:**
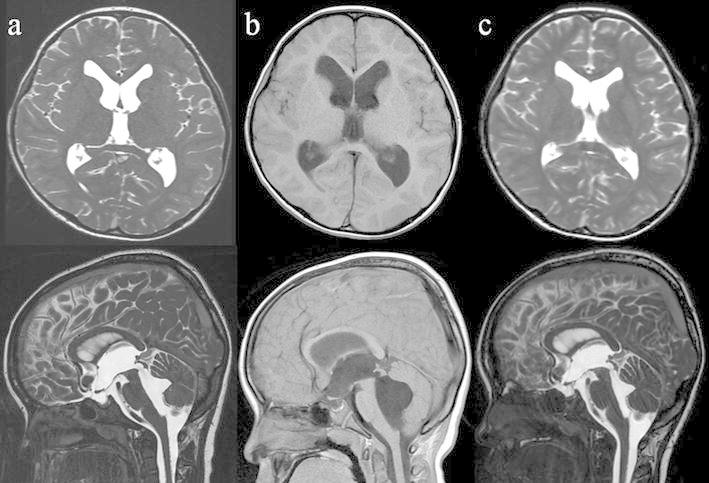
**a** Magnetic resonance imaging (MRI; fast imaging employing steady state acquisition, FIESTA), performed 1 month after the surgery, shows significant shrinkage of each ventricle. **b** MRI (T1-weighted image), performed 1 year after the initial endoscopic third ventriculostomy (ETV), indicating recurrent hydrocephalus. **c** MRI (FIESTA), performed 6 months after the second ETV, indicating improvement of the hydrocephalus

A 3-year-old boy without any medical history presented with a headache and vomiting and was referred to our institute. Computed tomography (CT) images, which had incidentally been taken 2 years before at a local neurosurgical clinic for the assessment of a minor head injury, were available and showed no significant abnormality (Fig. 1a). The patient had no neurological deficit on admission. Magnetic resonance imaging (MRI) showed enlargement of all ventricular systems associated with the dilatation of the foramina of Magendie and Luschka, with no obstructive organic lesions (such as brain tumors) on contrast enhanced MRI (Fig. 1b–f). The patient underwent emergent ventricular drainage through the anterior horn of the right lateral ventricle to relieve his symptoms. We had initially considered this case might be a communicating hydrocephalus because the fourth ventricle outlets appeared to be patent on MRI. However, the highly expanded fourth ventricle and its outlets were inconsistent with communicating hydrocephalus. We thus conducted CT ventriculo-cisternography on the fifth day post-surgery by injecting contrast medium (Isovist^®^) via a ventricular catheter (Fig. 2). On serial CT images, contrast medium was accumulated in the foramina of Magendie and Luschka 1 h after injection, with limited diffusion to the adjacent cisterns at 3 h after injection. These findings suggested a mechanical obstruction at the outlets of the fourth ventricle. We therefore diagnosed the patient with FVOO and chose to perform an ETV using a flexible neuro-endoscope (VEF-V, Olympus, Tokyo, Japan). The endoscope was inserted into the fourth ventricle through the dilated cerebral aqueduct and encountered the suspected cause of obstructive hydrocephalus—a thickened, arachnoid, membranous structure that enveloped the foramina of Magendie and Luschka (Fig. 3). A standard third ventriculostomy in the tuber cinereum was performed without any additional surgical intervention to the membranous structures in the fourth ventricle outlets. On endoscopic observation of the lateral ventricle and pre-pontine cistern, no abnormalities suggestive of previous meningitis or intraventricular hemorrhage were found. The patient’s postoperative course was uneventful with no signs of neurological deficit. MR images obtained 1 month post-surgery revealed significant resolution of the hydrocephalus (Fig. 4a). However, after 1 year, the patient again presented with vomiting, and it was noted that the hydrocephalus had recurred (Fig. 4b). Re-exploration with the endoscope revealed severe stenosis of the fenestration site from the first surgical procedure and a second ETV was subsequently performed. Postoperative MRI confirmed that the hydrocephalus was resolved (Fig. 4c). The patient has remained in good condition without recurrence of hydrocephalus, as identified by MRI, since undergoing the second ETV for 20 months.

## Discussion

FVOO is a rare cause of obstructive hydrocephalus. Although many studies on FVOO have been published, the pathogenic mechanism of this condition remains unclear. FVOO tends to occur in children and may be congenital (Inamura et al. 2002; Rifkinson-Mann et al. 1987; Takami et al. 2010), but adult cases are also in fact common. There have been no reports that gender affects the prevalence of this condition.

### Idiopathic FVOO

Mohanty et al. (2008) reported a case series of 22 patients with FVOO; of these, 10 patients had a medical history, 3 had suffered intraventricular hemorrhage, and 7 patients had infections, including tubercular meningitis, bacterial infection, or prolonged and unexplained fever. The present case had no such obvious medical histories. In addition, the CT images that had incidentally taken 2 years prior showed no abnormality. Although head injury can be a cause of membranous obstruction of CSF, the injury of this case was quite minor without showing any intracranial hemorrhage or brain contusion on the CT images. These facts suggest that the present case represents a case of ‘idiopathic’ FVOO. There has been only one reported case thus far of acquired FVOO, in which previous radiological studies provided evidence of normal-sized ventricles (Suehiro et al. 2000).

### Diagnostic modalities for FVOO

The pattern of ventriculomegaly in FVOO is termed as panventriculomegaly (Mohanty et al. 2008) or tetra-ventricular hydrocephalus (Longatti et al. 2009; Takami et al. 2010), as dilatation occurs in both lateral ventricles as well as in the third and fourth ventricles (Huang et al. 2001; Karachi et al. 2003; Oertel et al. 2010; Roth et al. 2012; Suehiro et al. 2000). Dilatation or large CSF collection of the foramina of Magendie and Luschka is a characteristic radiological finding in cases of FVOO (Huang et al. 2001; Karachi et al. 2003; Roth et al. 2012; Takami et al. 2010). However, it is difficult to confirm the presence of a membranous obstruction via conventional MRI (Dincer et al. 2009). High-resolution three-dimensional constructive interference with steady state sequence on 3T MRI may be able to detect obstructive membranes (Dincer et al. 2009), although this may not be possible in all cases (Oertel et al. 2010). The most sensitive diagnostic method is CT ventriculography, with the injection of contrast medium through a ventricular catheter (Rifkinson-Mann et al. 1987; Roth et al. 2012). Serial CT images after injection will show collected contrast medium in the outlets of the fourth ventricle and subsequent blockage of its diffusion to the pre-pontine cistern. One concern about this method is radiation exposure of the brain, particularly in younger children. Joseph et al. (2003) recommended the use of MRI instead of CT as the diagnostic modality for FVOO in order to avoid exposure to radiation. As alternative examination to access the dynamics of CSF, efficacy of phase-contrast MRI (Choi et al. 1999; Huang et al. 2001), cine-MRI (Carpentier et al. 2001; Choi et al. 1999; Hashimoto et al. 2014; Inamura et al. 2002; Karachi et al. 2003; Longatti et al. 2009; Suehiro et al. 2000) or radioisotope cisternogram (Choi et al. 1999; Suehiro et al. 2000) is also reported.

Another diagnostic option is direct endoscopic inspection of the fourth ventricle in case where the aqueduct is sufficiently expanded to safely insert a neuro-endoscope through it (Mohanty et al. 2008). Although this technique needs to be done under general anesthesia and carries a risk of damaging the midbrain around the aqueduct, it has recently been reported to be relatively safe (Longatti et al. 2005, 2006; Mohanty et al. 2008; Torres-Corzo et al. 2014). When FVOO is highly suspected solely with MRI, this technique could allow simultaneous diagnosis and treatment, thereby reducing the chance of radiation exposure, duration of hospitalization, and risk of drainage infection.

### Treatment options of FVOO

Although a ventriculo-peritoneal (V-P) shunt is the most conventional treatment for FVOO (Longatti et al. 2009), it is not in fact preferable in children, who represent the majority of patients. In the past, direct fenestration of the membranous occlusion through craniotomy was attempted for treating FVOO (Longatti et al. 2009; Mohanty et al. 2008); however, recent studies have suggested that ETV is a less invasive and effective treatment strategy (Ferrer and de Notaris 2013; Hashimoto et al. 2014; Longatti et al. 2009; Mohanty et al. 1999, 2008; Oertel et al. 2010; Suehiro et al. 2000). Therefore, correct preoperative diagnosis is very important because ETV can eliminate the need for surgical implantation of a V-P shunt. In the imaging study by Dincer et al. (2009) with the 3D-CISS sequence on 3T MRI, they found 26 endoscopically treatable noncommunicating cases among 134 cases who had been previously diagnosed as communicating hydrocephalus by conventional MR images.

We conducted systematic review of previous case series and reports to assess the efficacy of ETV for FVOO. English articles were identified via a PubMed search using the key words “fourth ventricle outlet obstruction”, “fourth ventricular outlet obstruction”, “fourth ventricle outflow obstruction”, “FVOO”, “membranous obstruction of the fourth ventricle”, “primary obstruction of the fourth ventricle”, “obstruction of Magendie’s and Luschka’s foramina”, “obstruction of fourth ventricular exit” or “far distal obstructive hydrocephalus”. From these search results, we identified 9 articles that included the case of FVOO treated solely by ETV (Table 1). All references in these papers were also screened.

**Table 1:** Summary of case reports or series of FVOO treated only by ETV

References	Number of cases	Primary/secondary	Diagnostic modalities	Endoscopic exploration of fourth ventricle	Number of ETV success	Rate of ETV success (%)
Choi et al. (1999)	2	Not regarded	CT Radioisotope cisternogram Phase-contrast MRI Cine-MRI	No	2	100
Mohanty et al. (1999)	3	Primary	CT MRI Ventriculography	No	3	100
Suehiro et al. (2000)	1	Primary	MRI Cine-MRI Radioisotope cisternogram	No	1	100
Carpentier et al. (2001)	1	Primary	MRI Cine-MRI	No	1	100
Karachi et al. (2003)	3	Primary	MRI Cine-MRI Ventriculography	No	3	100
Mohanty et al. (2008)	22 (20)^a^	Primary (12 cases) and secondary (10 cases)	CT MRI Ventriculography	Yes	13	65
Longatti et al. (2009)	8	Primary	CT MRI Cine-MRI	Yes	6^b^	75
Oertel et al. (2010)	4^c^	Secondary	MRI	Yes	2	50
Hashimoto et al. (2014)	1	Primary	CT MRI Cine-MRI Ventriculography	No	1	100
Present case	1	Primary	CT MRI Ventriculography	Yes	1	100

*Idiopathic* FVOO without previous medical history, *secondary* FVOO with previous medical history such as meningitis or intracranial hemorrhage

^a^ETV was not performed because of technical difficulties in 2 of 22 patients

^b^One case was lost during the follow-up period

^c^The cases considered as FVOO among 20 cases of far distal obstructive hydrocephalus

Mohanty et al. (2008) reported the entire success rate of ETV for FVOO is 65 % (13 successes in 20 cases). Although they did not evaluate the success rates of primary and secondary FVOOs separately, they speculated that failure was attributable to CSF malabsorption as a result of prior meningitis or intraventricular hemorrhage. Oertel et al. (2010) reported surgical results of ETV in 20 cases of far distal obstructive hydrocephalus. Most of their cases were with Dandy Walker or Arnold Chiari malformations, however, there were four cases considered as secondary FVOO. Two of the four patients (50 %) were successfully treated by ETV, while remaining two patients required early shunting. In contrast, other case reports or case series (Carpentier et al. 2001; Hashimoto et al. 2014; Karachi et al. 2003; Longatti et al. 2009; Mohanty et al. 1999; Suehiro et al. 2000) of ETV performed exclusively for primary FVOO demonstrated obviously better outcome (75–100 %). According to these previous reports, ETV would be more effective in patients of primary FVOO.

Mohanty et al. (2008) described that most failures of ETV for treating FVOO occur within 6 weeks of surgery and that subsequent endoscopic re-exploration revealed patency at the fenestration site. The recurrent hydrocephalus in our case differs from that report in two aspects: first, recurrence occurred 1 year after the initial ETV, which was considerably delayed when compared with those reported cases; second, endoscopic exploration not only confirmed a highly stenosed fenestration site, but re-expansion of the fenestration also relieved the hydrocephalus. Indeed, this suggests that the recurrence of hydrocephalus observed in our case is not attributable to CSF malabsorption. In such cases (where recurrent hydrocephalus does not appear to be caused by malabsorption), repeated ETV would be an effective treatment option.

Endoscopic foraminoplasty by direct fenestration of membranous obstruction at the fourth ventricle outlets is another previously reported treatment option (Longatti et al. 2006, 2009; Torres-Corzo et al. 2014). However, its usefulness is still unclear because it was used in combination with ETV in most cases. Although there were some reports with good outcome solely by this technically more demanding procedure, we believe it must be considered only when ETV is difficult or ineffective for some reasons and must be performed by well-experienced neuroendoscopists.

## Conclusion

In the present report, we describe a case of idiopathic FVOO with no remarkable medical history that resulted in the development of hydrocephalus. ETV should be considered as a treatment option for FVOO, particularly in idiopathic cases without CSF absorption disorders. Moreover, in such cases, endoscopic re-exploration of the fenestration would be effective, even when hydrocephalus recurs.

## Consent

Written informed consent was obtained from patient’s parents for the publication of this study and accompanying images.

## Abbreviations

CSF: cerebrospinal fluid
CT: computed tomography
ETV: endoscopic third ventriculostomy
FVOO: fourth ventricle outlet obstruction
MRI: magnetic resonance
V-P: ventriculo-peritoneal
